# Aberrant spindle dynamics and cytokinesis in *Dictyostelium discoideum* cells that lack glycogen synthase kinase 3^☆^

**DOI:** 10.1016/j.ejcb.2013.05.001

**Published:** 2013-06

**Authors:** Adrian J. Harwood, Josephine E. Forde-Thomas, Hazel Williams, Matthias Samereier, Annette Müller-Taubenberger

**Affiliations:** a School of Biosciences, Cardiff University, Museum Ave., Cardiff CF10 3US, United Kingdom; b Institute for Anatomy and Cell Biology, Ludwig Maximilian University of Munich, Schillerstr. 42, 80336 Munich, Germany

**Keywords:** Glycogen synthase kinase 3, Mitosis, Cytokinesis, Mitotic spindle, *Dictyostelium*

## Abstract

Eukaryotic cell division requires the co-ordinated assembly and disassembly of the mitotic spindle, accurate chromosome segregation and temporal control of cytokinesis to generate two daughter cells. While the absolute details of these processes differ between organisms, there are evolutionarily conserved core components common to all eukaryotic cells, whose identification will reveal the key processes that control cell division. Glycogen synthase kinase 3 (GSK-3) is a major protein kinase found throughout the eukaryotes and regulates many processes, including cell differentiation, growth, motility and apoptosis. In animals, GSK-3 associates with mitotic spindles and its inhibition causes mis-regulation of chromosome segregation. Two suppressor screens in yeast point to a more general effect of GSK-3 on cell division, however the direct role of GSK-3 in control of mitosis has not been explored outside the animal kingdom. Here we report that the *Dictyostelium discoideum* GSK-3 orthologue, GskA, associates with the mitotic spindle during cell division, as seen for its mammalian counterparts. *Dictyostelium* possesses only a single GSK-3 gene that can be deleted to eliminate all GSK-3 activity. We found that *gskA*-null mutants failed to elongate their mitotic spindle and were unable to divide in shaking culture, but have no chromosome segregation defect. These results suggest further conservation for the role of GSK-3 in the regulation of spindle dynamics during mitosis, but also reveal differences in the mechanisms ensuring accurate chromosome segregation.

## Introduction

Glycogen synthase kinase 3 (GSK-3) is a serine-threonine protein kinase that was originally identified as a key regulator of insulin-dependent glycogen synthesis (Woodgett, 1990). GSK-3 has subsequently been shown to function in a wide range of signalling pathways including the Wnt, Hedgehog and Notch signal transduction pathways, protein synthesis and apoptosis (for a review see Forde and Dale, 2007). Several studies have linked GSK-3 to the regulation of microtubule dynamics. GSK-3β has been shown to phosphorylate several microtubule-associated proteins (MAPs), including CLASP2 (Watanabe et al., 2009), Collapsin Response Mediator Proteins (CRMPs; Cole et al., 2004), adenomatous polyposis coli (APC; Zumbrunn et al., 2001), Tau (Lovestone et al., 1996), MAP2C (Sanchez et al., 2000) and MAP1B (Goold et al., 1999). Each of these MAPs influence microtubule stability though direct interactions with microtubules, and in each case GSK-3 mediated phosphorylation was found to decrease their ability to stabilize microtubules.

Wakefield and co-workers reported that mammalian GSK-3β localized specifically to mitotic microtubules and centrosomes in HeLa cells (Wakefield et al., 2003). Inhibition of mammalian GSK-3 with chemical inhibitors caused defects in astral microtubule length, chromosome alignment and an increase in the frequency of micronuclei (Mishima et al., 2008; Tighe et al., 2007; Wakefield et al., 2003). In *C. elegans*, loss of GSK-3 interferes with mitotic spindle orientation in 4-cell stage embryos (Schlesinger et al., 1999), and in *D. melanogaster*, GSK-3β localizes to the centrosome (Bobinnec et al., 2006) and loss of function mutations cause aberrant mitotic spindle morphology (Wojcik, 2008). Although these results suggest that there may be a conserved role of GSK-3 controlling spindle function in animal cells, there have been no reports of regulation of spindle dynamics or cytokinesis outside the animal taxon. However, a number of yeast suppressor screens suggest that a link between GSK-3 and mitotic control may be more fundamental. In *S. cerevisiae*, deletion of one of the four yeast GSK-3 orthologues, MCK1, suppressed the effects of some classes of centromere DNA mutation, caused a rise in the rate of mitotic chromosome loss and increased sensitivity to the microtubule destabiliser benomyl (Shero and Hieter, 1991). In *S. pombe*, over-expression of the GSK-3 orthologue Skp1 suppresses the cytokinesis mutant cdc14 (Plyte et al., 1996). Prompted by these earlier reports, we carried out a characterization of *Dictyostelium discoideum*, where disruption of its GSK-3 orthologue *gskA* eliminates all GSK-3 activity.

In *Dictyostelium*, the processes of chromosome segregation and cell division have been well characterized. During mitosis, an intra-nuclear spindle forms, elongates and separates chromosomes, and finally disassembles at the mid-zone (Effler et al., 2006; Neujahr et al., 1998; Roos and Camenzind, 1981). Spindle dynamics are coupled to other mitotic events, for example chromosome attachment, anchorage of aster microtubules to the cell cortex and positioning of the cleavage furrow (Neujahr et al., 1998; Niewöhner et al., 1997).

*Dictyostelium* cells express only a single GSK-3 homologue, GskA. Expression of GskA is not essential for cell survival (Harwood et al., 1995). However, as these cells enter development *gskA* null cells exhibit abnormalities: aggregation territories are greatly reduced; cells are chemotaxis defective and do not stream, but rather form small loose mounds in a random and disordered manner; slugs migrate shorter distances and fruiting bodies develop with an enlarged basal disc and small spore head (Harwood et al., 1995; Teo et al., 2010). *gskA* null cells also exhibit altered gene expression patterns (Schilde et al., 2004; Strmecki et al., 2007).

Here, we report that GskA localizes to the mitotic spindle and that *gskA* null cells exhibit defects in spindle assembly and orientation. When grown in shaking culture, *gskA* null cells exhibit a defect in cytokinesis. However, we observe no defect in chromosome segregation. These results indicate a partially conserved role for GSK-3 in mitosis to coordinate spindle dynamics during early prometaphase.

## Results and discussion

### Localization of GskA-GFP in Dictyostelium

**Fig. 1:**
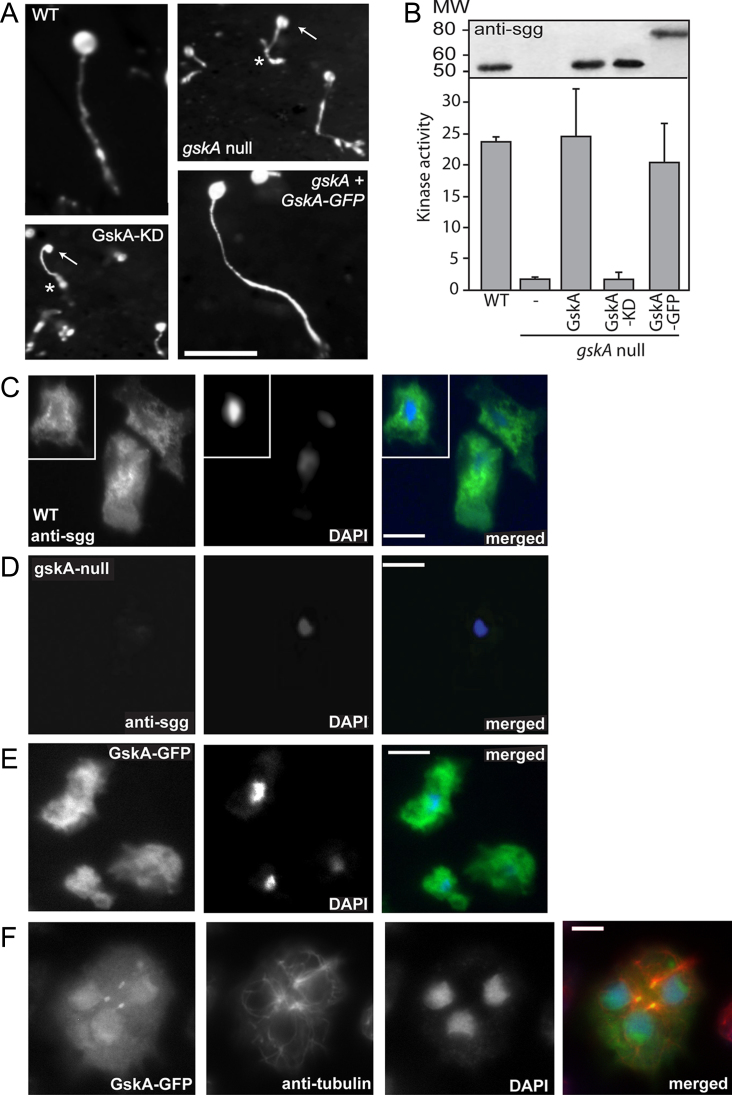
(A) GFP-GFP restores GskA function. *gskA* null cells exhibit developmental defects leading to an aberrant fruiting body morphology. Terminally differentiated *Dictyostelium* wild-type cells, *gskA* null cells and *gskA* null cells expressing either a kinase dead (KD) GskA^K85R^ mutant or the GskA-GFP were imaged 24 h after plating on non-nutritive phosphate agar plates to induce development. Cells lacking active GskA have fruiting bodies that are significantly smaller and morphologically distinct. * indicates an enlarged basal disc, arrow indicates small spore head. Expression of GskA-GFP in *gskA* null mutants fully restores the wild type-like appearance of fruiting bodies. All photographs are at the same magnification, bar, 500 μm. (B) The GskA-GFP fusion is catalytically active. Kinase assays were performed to compare the catalytic activity of GskA in wild-type cells and *gskA* null cells expressing GskA, GskA-GFP or a kinase dead (KD) GskA^K85R^ mutant. To assess the level of background activity, *gskA* null cells were included in the assay. Kinase activity = pmol phosphate transferred/mg protein/min. Inset shows an anti-sgg, which recognizes GSK-3 proteins from all species, Western to demonstrate expression of the GskA and GskA-GFP proteins (C and D) anti-sgg, antibody detects GskA within the cytoplasm and nucleus of wild-type cells (C) but not in *gskA* null cells (D) in merged images, GskA is shown in green and DNA in blue. Figure C shows three cells, two clustered together and a third from a separate field (inset). (E) The pattern of GskA-GFP in transformed cells matches that seen with anti-sgg antibody. (F) Although during interphase, in most cells GskA-GFP is most abundant in the cytoplasm, in approximately 1% of cells, GskA-GFP is enriched in the nucleus and co-localized with structures with characteristics of centrosomes,, as determined by their enriched anti-α-tubulin staining as commonly seem in *Dictyostelium* (cells fixed with glutaraldehyde and immunolabeled using an antibody against α-tubulin). These cells were observed to rapidly proceed into mitosis. Bar, 5 μm.

*gskA* null mutants have a distinctive morphological phenotype, where cells culminate to form small, mis-proportioned fruiting bodies with enlarged basal discs, short stalks and reduced spore heads (Harwood et al., 1995; Fig. 1A). To examine the sub-cellular distribution and functional dynamics of GskA, we created GskA-GFP fusion genes and expressed them in wild type and *gskA* null mutant cells. Expression of GskA-GFP from an *actin15* promoter was sufficient to restore wild type development (Fig. 1A). Kinase assays confirmed that there was no GSK-3 kinase activity in *gskA* null mutant cells, but that re-expression of GskA from an *actin15* promoter restored wild type levels of GSK-3 activity (Fig. 1B). No restoration of activity was observed with a kinase-dead (KD) GskA-K85R mutant protein. Wild type levels of GSK-3 activity were observed in cells expressing a GskA-GFP fusion protein, consistent with its ability to rescue the *gskA* null mutant phenotype.

During interphase, GskA protein is localized throughout the cell, being present in both cytoplasm and nucleus. No enrichment was observed at the cell cortex or membrane (Fig. 1C). Higher levels of GskA appeared to be present in the perinuclear cytoplasm and, although present, the concentration of GskA in the nucleus in most cells appeared lower than in the cytoplasm. Occasional cells were observed with slight nuclear enrichment of GskA protein as judged by antibody staining (data not shown). A similar protein distribution was observed in cells expressing a GskA-GFP fusion protein, indicating that the presence of the GFP did not alter GskA protein distribution (Fig. 1E). GFP fluorescence was monitored as cells were starved and entered development; however the sub-cellular distribution of GskA-GFP remained constant throughout development and between cell types (data not shown). Again, a small number of cells (<1%) showed nuclear enrichment of GskA-GFP. In addition, these cells also showed enrichment on structures with the characteristics of *Dictyostelium* centrosomes (Fig. 1F; Daunderer et al., 1999; Schulz et al., 2009). Analysis of time-lapse videos showed that nuclear and centrosomal enrichment preceded (data not shown) and followed on from mitosis (movie 1).

**Fig. 2:**
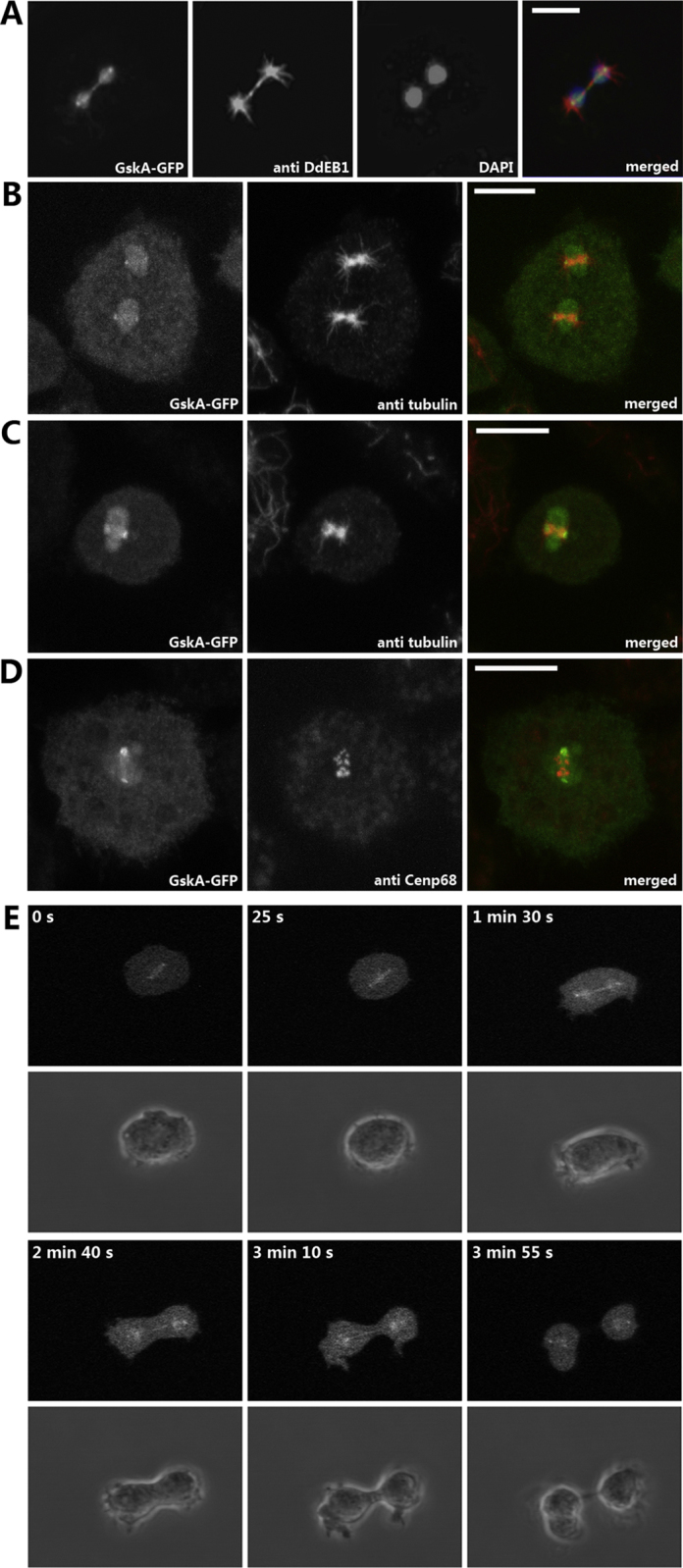
GskA-GFP associates with the mitotic spindle and remains enriched within the nucleus. GskA-GFP expressing cells were fixed with methanol (A), or glutaraldehyde (B, C, D), respectively, and were labelled with anti-DdEB1 (A), anti-α-tubulin (B, C), anti-Cenp68 (D) and DAPI (A). GskA-GFP was found to be associated with the spindle poles during all mitotic stages. Unlike several over-expressed nuclear GFP-fusion proteins that exit the nucleus during prometaphase, when the *Dictyostelium* nuclear envelope partially breaks down, GskA-GFP enriches inside the nucleus. At no point during mitosis was GskA-GFP observed to localize to kinetochores as analyzed by counterstaining of GskA-GFP cells with the centromere/kinetochore marker anti-Cenp68. (E) Live cell imaging experiments, confocal time series, of a mitotic GskA-GFP expressing wild-type cell, showing an enrichment of GskA at centrosomes, mitotic spindle and midbody Upper array shows GFP-fluorescence, the lower array the corresponding brightfield images. In merged images, GskA-GFP is shown in green, DAPI in blue (A), α-tubulin (A, B, C) or Cenp68 (D) in red. Bar, 5 μm.

As cells underwent division the sub-cellular distribution of GskA-GFP showed clear re-localization. As mitosis proceeds, GskA-GFP becomes localized along the central spindled, remaining associated until the spindle separated (Fig. 2). *gskA* null cells expressing GskA-GFP were fixed and stained for the microtubule binding protein Dd-EB1 (Rehberg and Gräf, 2002). During mitosis, Dd-EB1 was present on both central and astral microtubules as well as at the centrosomes. GskA-GFP co-localized with Dd-EB1 along the central spindle, but not along astral microtubules (Fig. 2A). Immunolabeling of wild-type cells expressing GskA-GFP with anti-α-tubulin antibodies confirmed the localization of GskA-GFP along the central spindle and centrosomes (Fig. 2B and C). Further immunostaining studies showed that GskA-GFP is absent from kinetochores of chromosomes during mitosis (Fig. 2D). Live-cell microscopy revealed that GskA-GFP relocates into the nucleus shortly before the onset of mitosis, and associates with the newly formed mitotic spindle, persisting until mitosis is completed (Fig. 2E, and movie 1).

Our observations in *Dictyostelium* show an association of GSK-3 with the mitotic spindle, as previously reported in HeLa cells (Wakefield et al., 2003). In the human case centrosome-associated GSK-3 was reported to be inhibited by phosphorylation on Ser21 of GSK-3α and Ser9 of GSK-3β respectively. In contrast, the *Dictyostelium* kinase lacks an equivalent phospho-regulatory site, and this suggests that GSK-3 inhibition via phosphorylation may not be a core requirement for its mitotic function. Indeed we note that no mitotic defects have been reported in mouse knock-in mutant cells that lack both Ser9/Ser21 phosphorylation sites of all GSK-3 proteins (McManus et al., 2005).

### gskA null cells exhibit defects in spindle elongation and orientation

**Fig. 3:**
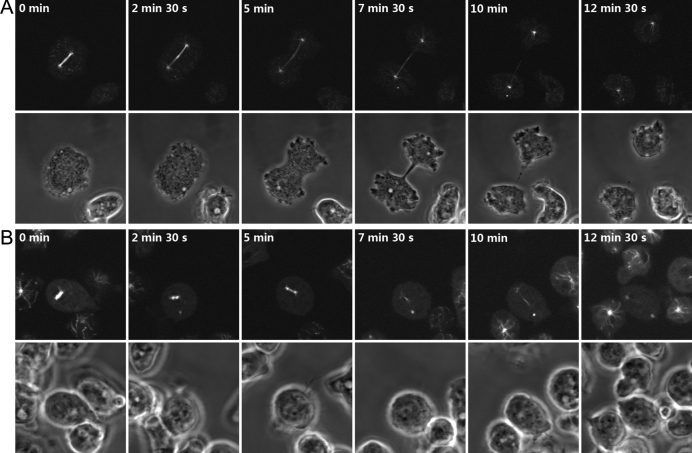
*GskA* null cells are retarded in prometaphase progression in comparison to wild type. Shown are still images of confocal time series of GFP-α-tubulin expressing wild-type (A), or *gskA* null cells (B) undergoing mitotic divisions. Spindle elongation and the transition of prometaphase to metaphase, was frequently observed to be impaired in the absence of GskA.

To investigate the potential role of GskA during cell division, a GFP-α-tubulin fusion protein was expressed in both wild type and *gskA* null cells. Expression of GFP-α-tubulin allowed visualization of the mitotic spindle dynamics in individual cells. In wild-type cells, the beginning of mitosis is characterized by disappearance of the interphase microtubule array. In prometaphase, the centrosome is duplicated and splits into two halves that are separated by about 1 μm (Gräf et al., 2004). The two spindle poles are connected by a thread of microtubules that form the mitotic spindle. After a lag period of 2–5 min (Tikhonenko et al., 2008) the spindle poles move apart from each other and the mitotic spindle elongates to a length of approximately 12–14 μm, this is accompanied by chromosome separation. In parallel, the cell cortex constricts in an actin-dependent manner, and finally the daughter cells are separated (Fig. 3A, movie 2). In contrast, in *gskA* null cells, the progression of spindle formation is retarded at an early stage and the spindle apparatus remains stalled in a dumbbell shape for a prolonged period of time. This failure of the central spindle to elongate means that in most cases (∼ 85%) the spindle poles are not separated in a conventional manner (Fig. 3B, and movie 3). In wild type *Dictyostelium* cells, the complete mitotic cycle is variable, in our analysis ranging from 2 to 20 min, due to variation in the timing of abscission to form the daughter cells in the final stages of mitosis. The *gskA* shows a much earlier mitotic block, commencing at pro-metaphase, and the eventual resolution of this block to form daughter cells also prolongs mitosis. However, given the different mechanism and timing of these sources of cycle length variation it is not possible to make an accurate comparison between wild type and *gskA* null cells cycle times.

### gskA null cells exhibit defects in cytokinesis

When grown on solid surfaces wild-type cells round up into a sphere at the onset of mitosis, but then flatten along the axis formed by the elongated spindle. Their spindles remain at a constant depth in the cell and consequently can be observed in a single focal plane. *gskA* null cells do not exhibit this behaviour; no cell flattening can be observed during division and only occurs in daughter cells after separation. As a consequence the position of the assembled but un-extended mitotic spindle is rarely fixed within the cell.

**Fig. 4:**
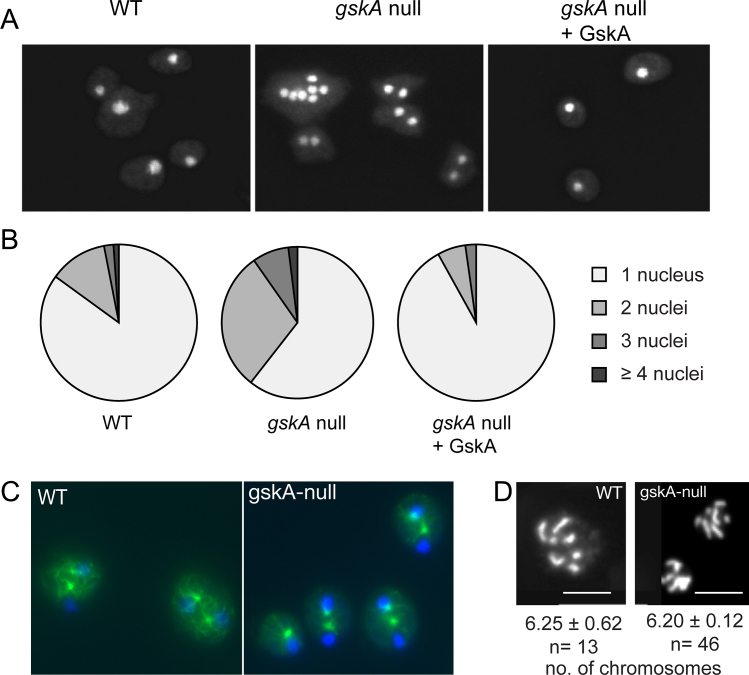
*gskA* null cells are defective in cytokinesis. When grown in shaking culture, gskA-null cells were found to exhibit a significantly increased number of multinucleated cells. (A) Representative images of wild-type cells (left), *gskA* null cells (centre), and *gskA* null cells expressing GskA-GFP (right). Expression of GskA-GFP in *gskA* null cells restored the wild-type phenotype with mainly mono-nucleated cells. (B) Quantification of experiments as described in (A), summarizing three independent experiments. (C) Wild type and *gskA* null cells were grown on a solid surface and then stained for DNA (DAPI) and microtubules (anti-α-tubulin). (D) Mitotic spreads were generated for wild type and *gskA* null cells, and numbers of chromosomes were counted for each cell (mean ± standard deviation).

In light of these observations, we looked more closely at the phenotype of *gskA* null cells during division. In *Dictyostelium* it is often the case that mitotic defects that do not actually prevent cell division when cells are grown on a solid surface have a stronger phenotype when cells are grown in shaking suspension. We found that the number of multinucleated cells is strongly increased in *gskA* null cells compared to wild-type cells (Fig. 4A and B). When these multinucleate cells were returned to a solid surface they underwent cytokinesis back into uni-nuclear cells. Expression of wild-type GskA-GFP was able to rescue this phenotype (Fig. 4A) while GskA^K85R^-GFP expression could not (data not shown). We propose that prolongation of spindle elongation increases the probability that cells will fail to undergo normal cytokinesis and produce a bi- or multinucleated cell in non-adherent conditions.

### gskA null cells are able to segregate their DNA

In mammalian cells, it was reported that GSK-3 inhibition causes both spindle defects, as seen in *gskA* null mutant cells, and problems with chromosome segregation, with approximately 60% of chromosomes failing to align at the spindle centre. To investigate whether this was the case in *Dictyostelium, gskA* null cells were fixed and stained cells with DAPI. In both wild-type cells and *gskA* null cells nuclear DNA accumulates at each of the spindle poles prior to cytokinesis, albeit more slowly in the case of gskA null cells (Fig. 4C). This suggests that loss of *gskA* does not grossly affect chromosome segregation and nuclear division. To investigate whether there may be subtle effects leading to mis-segregation of one or two chromosomes (aneuploidy), we prepared and examined mitotic spreads for both wild type and *gskA* null cells. We found no evidence for mis-segregation of chromosomes in the daughter cells (Fig. 4D). We conclude that in *Dictyostelium* GskA primarily regulates spindle dynamics, but not chromosome segregation.

## Concluding remarks

We have established that GSK-3 in *Dictyostelium* is associated with the mitotic spindle and is required for the normal progression of mitosis. This finding in many ways mirrors that of other studies reporting a spindle association and function of GSK-3 during mitosis, and suggests an evolutionary conserved role for GSK-3 (Mishima et al., 2008; Tighe et al., 2007; Wakefield et al., 2003). However, in contrast to those other reports, we find no evidence for a defect in chromosome segregation. Instead, we find that *gskA* null mutant cells have a defect in cytokinesis, leading to multinuclear cells when grown in shaking suspension. This may not be totally unexpected, as chemical or siRNA inhibition of GSK-3 also delays exit from mitosis in HeLa cells (Tighe et al., 2007), and in *S. pombe* over-expression of Skp1 suppresses a cytokinesis phenotype arising from loss of the protein phosphatase cdc14 (Plyte et al., 1996).

In mammalian cells, GSK-3 phosphorylates a number of microtubule-associated proteins (MAPs), including CLASP2 (Watanabe et al., 2009), APC (Zumbrunn et al., 2001), Tau (Lovestone et al., 1996), MAP2C (Sanchez et al., 2000) and MAP1B (Goold et al., 1999). Of these, APC, CRMP4 and CLASP2 have been linked to mitosis. Loss of either APC or CRMP4 can cause chromosome segregation defects (Fodde et al., 2001; Ong Tone et al., 2010). Interestingly neither of these MAPs appears to be present in *Dictyostelium*, and we predict the presence of a GSK-3-independent mechanism for maintaining correct chromosome segregation during *Dictyostelium* mitosis. In contrast, a CLASP orthologue has been identified from the *Dictyostelium* genome (DDB_G0271286). The function of this protein during mitosis has not been studied, but preliminary bioinformatic analysis suggests the presence of an EB1 protein site with embedded GSK-3 phosphorylation sites as observed in animal orthologues (Kumar et al., 2012; data not shown). Consistent with an effect via EB1, *Dictyostelium* mutants lacking this gene have a similar mitotic phenotype to that of the *gskA* null cells described here (Rehberg and Gräf, 2002).

CLASP proteins are plus-end tracking proteins (+TIPS) involved in the regulation of microtubule dynamics. CLASPs are mainly associated with microtubules via EB1 through a region of positively charged residues (SXIP). GSK-3 phosphorylation of this region suppresses the CLASP–EB1 interaction, and hence negatively regulates CLASP association with microtubule ends (Kumar et al., 2012; Watanabe et al., 2009). Interestingly, both CLASP and EB1 localize to the kinetochore (Maiato et al., 2004), a region where we see no GSK-3 present in *Dictyostelium*. We speculate that one function of GSK-3 is to restrict the interaction between CLASP and EB1 to kinetochore regions by blocking other possible interactions along the mitotic spindle. As cyclin dependent protein kinase 1 (CDK1) is the priming kinase for GSK-3 phosphorylation of CLASP2 this may provide cell cycle regulation of these interactions. Although less effective, CDK1 phosphorylation alone still weakens the CLASP–EB1 interaction (Kumar et al., 2012) and this could explain why loss of GSK-3 retards, but does not absolutely block, spindle extension. Finally, CLASP2, EB1 and GSK-3 additionally co-localize at the centrosome, however the functional significance of this interaction is less apparent.

In conclusion, we show that as seen in animals and suggested for yeast, *Dictyostelium* GSK-3 is also involved in regulating mitosis. This regulation is likely to be complex and involve multiple roles. Here we show that in *Dictyostelium* some, but not all, of these function are likely to be evolutionarily conserved, and may offer a simplified molecular genetic cell model to dissect these core functions in future studies.

## Materials and methods

### *Dictyostelium discoideum* strains and cell culture

*Dictyostelium discoideum* strains AX2 or DH1 are referred to as wild-type strains, and *gskA* null strains derived from these strains were used as described previously (Harwood et al., 1995; Teo et al., 2010). Cells were grown axenically at 22 °C in HL5 medium (Formedium). Development was assayed on KK2 agar plates. For expression of GskA-GFP different constructs were cloned and expressed in wild type or *gskA* null cells as indicated. The full-length coding sequence of gskA was cloned either in pDEX27 (Müller-Taubenberger, 2006), or into a vector allowing expression of GskA-superfolderGFP (Müller-Taubenberger and Ishikawa-Ankerhold, 2013). Alternatively, GskA-GFP2 was used (Teo et al., 2010).

### Immunostaining and live cell microscopy

For immunolabeling, wild type or *gskA* mutant cells settled onto glass coverslips were fixed with 15% picric acid/2% formaldehyde in 10 mM PIPES, pH 6.0, for 20 min and post-fixed with 70% ethanol for 10 min, or with methanol, and incubated with anti-EB1 (Rehberg and Gräf, 2002) or anti-sgg antibodies (Upstate) Immunofluorescence preparations to label centromeres using anti-Cenp68 (Schulz et al., 2009) or α-tubulin using YL 1/2 (Wehland and Willingham, 1983) antibodies, respectively, were conducted as described previously (Schliwa and van Blerkom, 1981). Briefly, cells adhering to glass coverslips and were fixed in half-concentrated PHEM buffer (30 mM PIPES, 12.5 mM HEPES, 5 mM EGTA, 1 mM MgCl_2_, pH 6.9) freshly supplemented with 0.25% glutaraldehyde and 0.25% Triton X-100 for 5 min. Unreacted glutaraldehyde was quenched with 1 mg/ml of freshly dissolved sodium borohydrate for 10 min prior to subjecting cells to immunolabeling. Nucleic acids were stained with DAPI (4′,6′-diamidino-2-phenylindole). From chromosome spreads cells were grown on acid-washed glass coverslips and treated with 33 μM nocodazole for 2 h. Cells were fixed in ice-cold ethanol:acetic acid (3:1) for 1 h and stained in 3 μl of mountant (Vectorshield; laboratories) containing 10 μg/ml DAPI (King and Insall, 2003).

Live-cell time series and fixed cell preparations were imaged using either an inverted LSM 410 or 510 Meta confocal microscope (Zeiss) equipped with 100× or 63× Neofluar 1.3 oil-immersion objectives. Images were taken using 488-nm argon and 543-nm helium-neon lasers in combination with 505–530 nm and 585–620 nm band-pass filters, respectively. Time series were recorded using 10–20 s intervals as indicated in the corresponding legend.

### Kinase assay

GSK-3 kinase activity in *Dictyostelium* cell extracts was determined using the GSK-specific peptide substrate, GSM, as described in Ryves et al. (1998). Briefly, cell extracts were incubated with ^32^P-γATP and GSM peptide, and the resulting phosphorylated peptide captured on P81 phosphocellulose paper. Phosphate incorporation was measured in the linear range at room temperature as picomoles of phosphate transferred to GSM peptide in 8 min.

## Appendix A Supplementary data

Supplemental Movie 1 Cytokinesis in a cell expressing GskA-GFP. GskA-GFP localizes to the mitotic spindle.

Supplemental Movie 2 Cytokinesis visualized by expression of GFP-α-tubulin in an AX2 wild-type cell.

Supplemental Movie 3 Cytokinesis visualized by expression of GFP-α-tubulin in a *gskA* null cell.
