# Supplementary figures and images for: Aberrant spindle dynamics and cytokinesis in Dictyostelium discoideum cells that lack glycogen synthase kinase 3
Source: Eur J Cell Biol. 2013 Jun;92(6-7):222–8. doi: 10.1016/j.ejcb.2013.05.001 (PMC3776220; doi:10.1016/j.ejcb.2013.05.001)

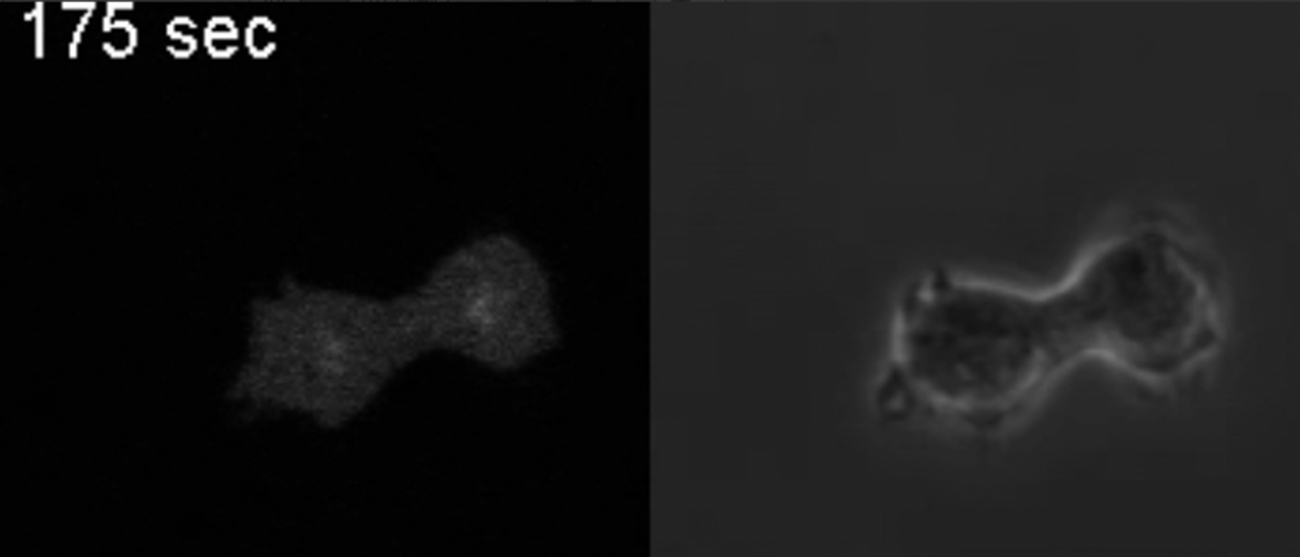

Supplement: Supplemental Movie 1 — Cytokinesis in a cell expressing GskA-GFP. GskA-GFP localizes to the mitotic spindle. [file mmc1.jpg]

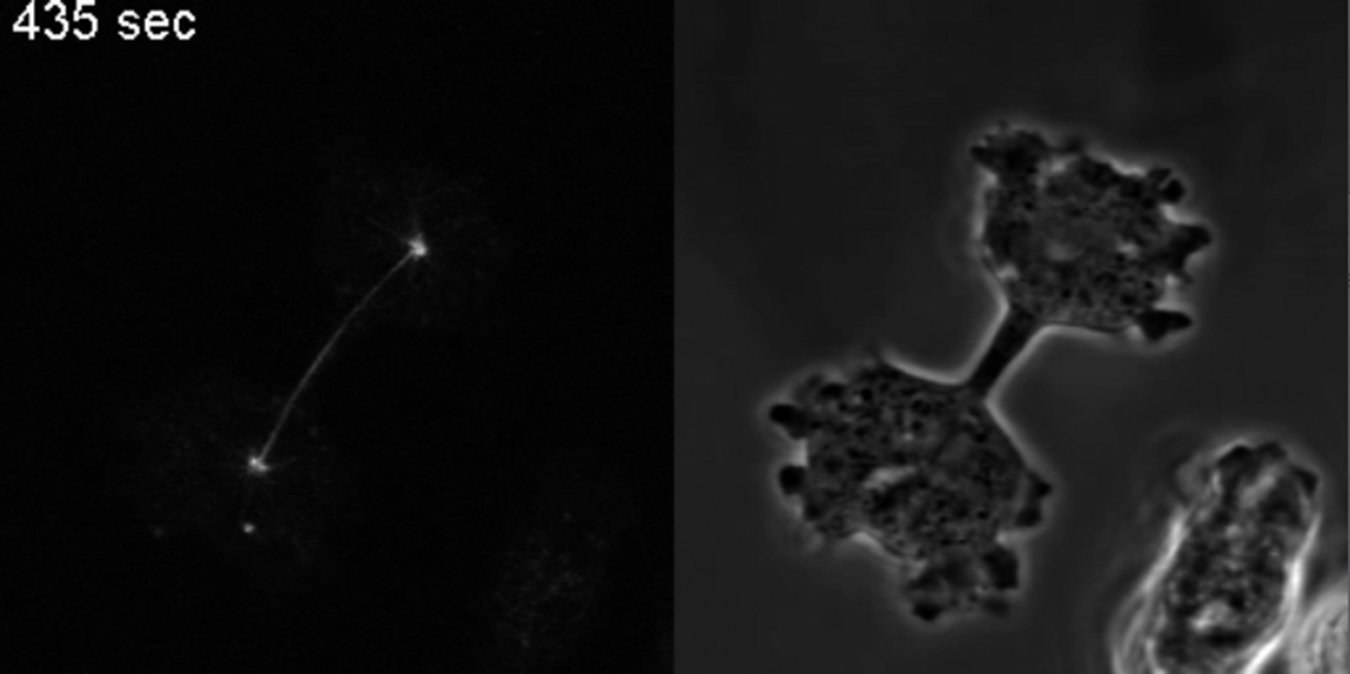

Supplement: Supplemental Movie 2 — Cytokinesis visualized by expression of GFP-α-tubulin in an AX2 wild-type cell. [file mmc2.jpg]

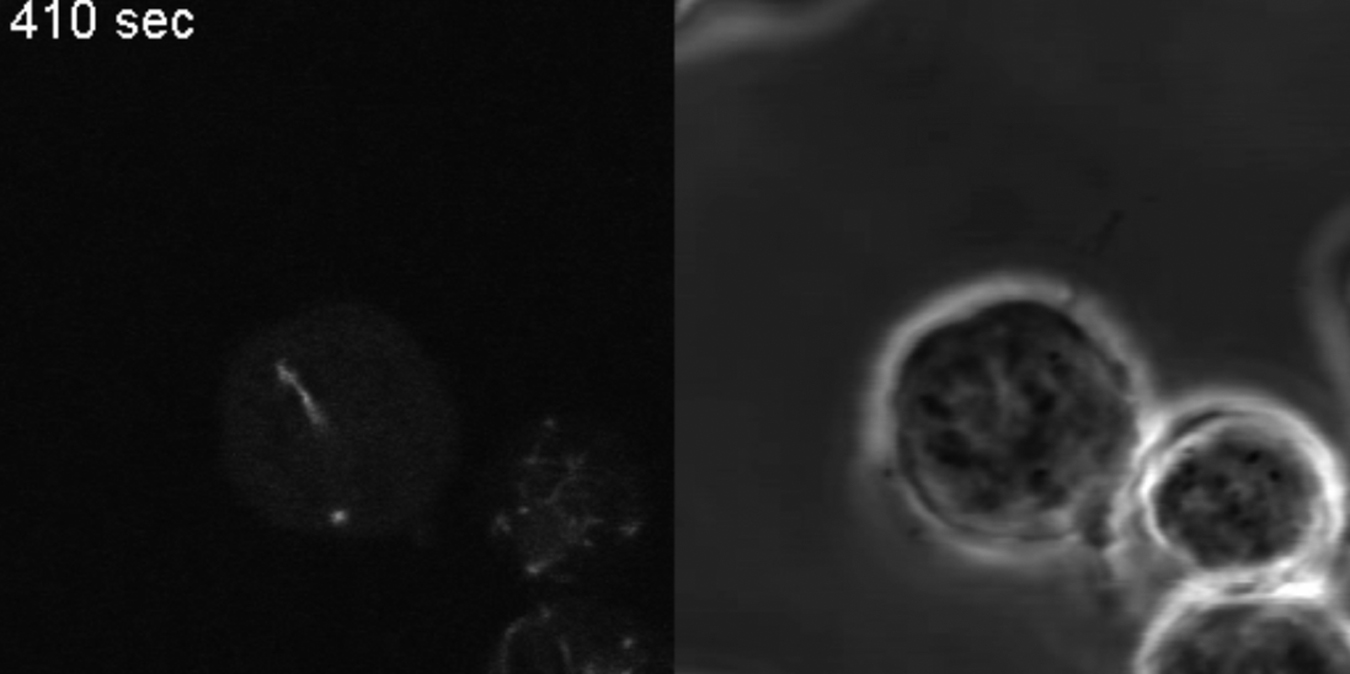

Supplement: Supplemental Movie 3 — Cytokinesis visualized by expression of GFP-α-tubulin in a gskA null cell. [file mmc3.jpg]
