# Supplementary material for: Aberrant spindle dynamics and cytokinesis in Dictyostelium discoideum cells that lack glycogen synthase kinase 3
Source: Eur J Cell Biol. 2013 Jun;92(6-7):222–8. doi: 10.1016/j.ejcb.2013.05.001 (PMC3776220; doi:10.1016/j.ejcb.2013.05.001)
Supplement: Supplementary file 4 [file mmc4.doc]

Ms. Ref. No.: EJCB-D-13-00006
Title: Aberrant spindle dynamics and cytokinesis in Dictyostelium discoideum cells that lack glycogen synthase kinase 3
European Journal of Cell Biology

| Received | Jan 17, 2013 |
| --- | --- |
| 1^st^ Decision | Feb 18, 2013 |
| 1^st^ Revision received | Apr 24, 2013 |
| 2^nd^ Decision | Apr 29, 2013 |
| 2^nd^ Revision received | May 06, 2013 |
| Accepted | May 07, 2013 |

**Decision letter**

Dear Prof. Harwood,

Many thanks for sending your manuscript to European Journal of Cell Biology. It has now been seen by two expert referees. You will see from the comments that both find merit in your paper. However, they also raise several important points, mainly related to figure quality and interpretation, as well as internal consistency, that preclude publication in the current form. Please address these points as closely as possible in a revision.  

Please submit a list of changes or a rebuttal against each point which is being raised when you submit the revised manuscript.

I am looking forward to a revised version of your manuscript.

Yours sincerely,

Stefan Linder
Editor

p.p. Dagmar Gebauer
Editorial Office
European Journal of Cell Biology

Reviewers' comments:

Reviewer 1

GSK3 has already been reported to have important functions in the regulation of mitotic spindle assembly and chromosome segregation and so on in organisms within the animal taxon, but there is no report of GSK3's roles in spindle dynamics or cytokinesis outside the animal taxon. Authors studied the roles of GSK3 using Dictyostelium and found that GSK3 associates with the mitotic spindle and is required for proper cytokinesis but not in chromosome segregation, arguing partial conservation of GSK3's roles in the regulation of spindle dynamics (cytokinesis) but differences in the mechanisms for chromosome segregation between Dictyostelium and other organisms.

    This report has significance in contributing the generalization of a scientific fact that GSK3 associates with mitotic spindle and is required for cytokinesis. I would give a recommendation of 'minor revision' for publish. However, there are several issues which must be satisfied since the presented data do not fully support the author's argues in the context.


<Major points>

Figure 1

Figure 1A.
page 5, middle: it says that 'fruiting bodies with enlarged basal discs, short stalks and small spore heads (Fig. 1A).'
    - However, in the figure 1A (gskA-null), it is very hard to see what is a basal disc or a spore. Additionally, the size of the spores could be different depending on the plane of focusing of microscope. According to the contexts in the introduction, these results in figure 1A have already been reported, so references should be added. Also, if there are markers (such as arrows) on the points in the figures, it would be easier to follow the context.

Figure 1C and D
- Upper panel in figure 1C is not necessary because it is repeated in the lower panel (figure 1C).

- Lower panel in figure 1C: the merged images would clearly show that GSKA colocalizes to the centrosomes and nuclei. Also, the letters such as GskA-GFP, anti-butublin, and DAPI in the figures should be colored with Green, Red, and Blue, respectively.
- page 5 line 2 from the bottom, 'but is enriched at centrosomes': It would be better to explain in more detail about why you think that.

Figure 1D (must be explained or corrected)
page 17 line 3 from the bottom: it says 'GSKA is distributed throughout the cytoplasm of wild-type cells and is in accordance with the localization of GskA-GFP.'
    - However, it is not true. Figure 1D shows that GskA is not evenly distributed in the cytoplasm and the merged images clearly show that GskA is not localized to the nucleus. These data conflict with those in figure 1C.


Figure 2

Figure 2A
- The colors of GskA, Dd-EB1, and DAPI are Green, Red, and Blue, respectively. The merged image in figure 2A must be explained in the legend. The merged image has red, yellow, blue, and purple colors and there is no green from GskA-GFP. How could this happen?

page 18 line 7, 'unlike several overexpressed nuclear.. GskA-GFP enriches inside the nucleus.'
    - However, I do not see which figures show these??

Figure 2D
page 6 line 17, it says 'GskA-GFP is absent from kinetochores'.
    - In my eyes, GskA-GFP colocalizes with Cenp68 since the merged image shows yellow colors as shown in the figure 2B and C indicating the colocalization of Gsk-GFP and tubulin. In order to tell the colocalization of Gsk-GFP and Cenp68, much higher resolution microscope should be used.


Figure 3

Page 8 in the middle, it says 'the assembled but unextended mitotic spindle is rarely seen in the center of the cell, and frequently shifts between focal planes, indicating that the spindle is unable to attach to the cell cortex and fix its position within the cell.'
    - I think that authors cannot say 'indicate'. There is no data in the paper indicating the relationship between the spindle and the cell cortex. Based on the presented data in figure 3, this conclusion is totally a kind of speculation so a little mild expression instead of 'indicating' should be used.

Figure 4

Page 9 line 5, 'In wild-type cells, nuclear DNA was seen to accumulate at each of the spindle poles prior to cytokinesis. In gskA null cells, two nuclei formed and segregated to each of the spindle poles, suggesting that the process of nuclear division and chromosome segregation was not grossly affected (Fig 4C)'
    - How could 'figure 4C' indicate the arguments like above? Please explain in more detail.

Page 9 line 10, 'but found no evidence for mis-segregation of chromosomes in the daughter cells (Fig. 4D).'
    - The experiments of mitotic spreads were performed on the cells grown on solid plates. According to this paper, there is no defect in cytokinesis and chromosome segregation when the cells were grown in the solid plates. Therefore, in my opinion the experiments of the mitotic spreads (counting chromosomes) should be done after shaking cultures and then expressed as the number of chromosomes/nucleus/cell since gskA null cells are multinucleated in shaking cultures.

<Minor points>

- gskA should be italic characters throughout the paper.

- Page 6 line6: (Data not shown) should be added at the end of the sentence.

- Page 11 line 3: Space between plates and For.

- Page 18 in the legend of fig2E, 'confocal time series taken of a mitotic wild type cell that expresses GSKA-GFP.' Should be a complete sentence.


Reviewer 2
Aberrant spindle dynamics and cytokinesis in Dictyostelium discoideum cells that lack glycogen synthase kinase 3 by Harwood et al is a descriptive study which attempts to analyze the role of the GSK3 analogue, GskA, during dictyostelium "development."  While the topic and cell choice are truly fascinating, excitement for this work is limited by the quality of the figures, the degree to which the reader is left guessing, the general sloppiness of the manuscript as a whole, and inconsistent and at times contradicting data.  For these reasons, which are summarized in the proceeding text, we recommend major revision of this manuscript.
Page 3 sentence 1:  This statement, albeit factually correct, requires a citation.
Page 3 paragraph 2 sentence 2:  The authors loosely use the word "inhibition" throughout the manuscript.  What type of inhibition - this needs to be clarified throughout the manuscript.  I.e. is this a small molecule drug, DNA/RNA approach, etc…
Page 5 paragraph 1 sentence 3:  This sentence needs to be clarified.  Transitioning from "coordinating spindle dynamics" to "impaired cytokinesis" leaves the reader confused.
Figure 1 : It does not appear that the images are at the same final magnificatation as indicated by the scale bar - in particular image 2. an inset of the nuclei (DAPI) for each image in Figure 1 A should include would confirm the authors position if true.
Figure 1 B:  An inset of the bands for the kinase assay should be included in lieu simply adding the quantitation.
Page 5 paragraph 3 sentence 1:  The authors state "…being present in both cytoplasm and nucleus, but is enriched at centrosomes (Fig. 1C)", the authors concluded that GskA-GFP is enriched at the centrosome, but use no known centrosomal marker; if the authors wish to conclude that GskA-GFP is at the centrosome, a centrosomal marker such as anti-gamma-tubulin or anti-pericentrin, as an overlay with GskA-GFP should be included.  Furthermore, it is unclear why the authors show an unrepresentative image with 3 nuclei.  This image, according to their Figure 4 B, only represents a small fraction of the normal phenotype, so why show something that is not representative?
Page 6 paragraph 2 sentence 1:  " In contrast, as cells underwent division the subcellular distribution of GskA-GFP showed clear re-localisation"  How do the authors know that this is not an artifact of the expression of GskA-GFP?  The authors only show 1 image of AX2 challenged with anti-GskA and state that this cell is in interphase.  If the authors wish to make a point about a change in localisation of GskA at various stages of the cell cycle then anti-GskA needs to be shown for each stage of the cell cycle for which a point is made.  Furthermore, the authors state that Figure 1 D illustrates that their expression method is consistent with the normal distribution of GskA, however, there appears to be a clear difference in the nuclear localization and level of expression as measured by fluorescence of anti-GskA compared to the GskA-GFP shown in Fig 1 C.
Page 6 paragraph 2 sentence 8: "and remains enriched in the nuclei of daughter cells…"  Can you confirm that these are nuclei and not spindle microtubules depolymerizing?
Page 6 paragraph 2 sentence 8: movie 1 does not show what the authors say it does;  Movie 1 shows spindle localization after the spindle has formed and further enrichment as division progresses.  
Page 6 paragraph 3 sentence 1: "association of GSK-3…"  HeLa cells have different isoforms of GSK.  The authors need to clearly state if this is pan-GSK that Wakefield et al tested.  Furthermore, the reviewer has evidence that GSK-3B is localized at the kinetochore region and midbody of hela cells, thus the authors overstate the agreement regarding localization of GSK.
Page 6 paragraph 3 sentence 1:  the authors need to fine-tune the use of the word respectively in this sentence.
Figure 2 E shows a complete cell cycle in 240 seconds, however, Figure 3 A demonstrates that a complete cell cycle take 10-12 minutes.  This is clearly not in agreement; it further prompts the question of how many times these experiments were conducted?  
Figure 3:  The authors are making a point about the time it takes to progress to cytokinesis.  However, the authors provide little evidence that these cells started at the same stage of the cell cycle.  The authors make a further point about the prometaphase stage but do not clearly show that these cells are actually at this stage.  A known marker for this stage needs to be employed in replicate samples.
Page 8 sentence 1: "…which is then followed by cytokinesis…(Fig. 3B, and movie 3)."  These data are not in agreement.  There is no evidence for cytokinesis.  In fact, the videomicroscopy approach employed as movie 3 does not show cytokinesis.
Page 8 paragraph 1 sentence 4:  "…mitotic spindle is rarely seen in the centre of the cell, and frequently shifts between focal planes, indicating that the spindle is unable to attach to the cell cortex and fix its position…"  This statement and interpretation is potentially problematic.  Since these cells were not immobilized the authors can not say that the spindle is moving and not the cell as a whole.  The investigators need to employ either a fiducial marker and make sure that the cell is not moving as a number of cells can be seen moving in and out of the field of view.  The argument that the spindle is in different focal planes is not valid.  Alternatively, the control cells should be grown in suspension and shown to contain a spindle in the same focal plane throughout the experiment.
Page 10 paragraph 1: " Interestingly neither of these MAPS…, and we predict the presence a GSK-3…"  an article (i.e. the word "of") is missing between the words "presence" and "a"
Page 10: "…co-localise at the kinetochore…"  there should be a citation here.
There is no clear pattern regarding spacing between paragraphs throughout this manuscript.
Page 11: "…was assayed on phosphate agar plates.For…:"  spacing.
Page 12: " 1mg/mL of freshly solved sodium..."  solved?  You mean dissolved?
Page 13: "…as described in Ryves et al, 1989. (Ryves et al…"   The period after 1989 belongs after the citation.
Page 17-19:  Figure legends typically should not include materials and methods, such as fixation protocol.  Furthermore,  "quantification of several experiments…"  several tells the reader very little.  It needs to be clearly stated how many independent experiments were performed

2^nd^ Decision

Dear Prof. Harwood,

Many thanks for sending the revised version of your manuscript. I am prepared to accept your paper, but would ask for clarification of a specific point.

In your reply to the question of reviewer 1 regarding Figure 1C you state that there are no markers like pericentrin available in Dictyostelium. Still, there are quite a few proteins that have been identified as part of the Dictyostelium centrosome such as CP224 (see publications of the Gräf group) or NdrA (by the Müller-Taubenberger group). These proteins could be used to verify the centrosomal localization of GskA, which is also mentioned on pp.6,7.

Many thanks for addressing this point.

Yours sincerely,

Stefan Linder
Editor

p.p. Dagmar Gebauer
Editorial Office
European Journal of Cell Biology

**Authors response letter**

(combined responses revision 1 and revision 2)

Please find a list of our revisions based on the comments of the two reviewers. We have addressed all points with revisions in the text and of Figures 1 and 4. We note however the comments about figure resolution. We find this puzzling, as the resolution of all of the images matches that requested by your journal and is the norm accepted by all other journals, perhaps there was an issue with the on-line copy for review. We have reviewed all of the figures, making any changes that were possible, and hope that these issues have now been resolved.

Specific revisions are outlined below (reply in blue).

Figure 1.

Both reviewers commented on Figure 1 and proposed revisions. We have fully revised this figure including new data where necessary.

Reviewer 1: However, in the figure 1A (gskA-null), it is very hard to see what is a basal disc or a spore. Additionally, the size of the spores could be different depending on the plane of focusing of microscope. According to the contexts in the introduction, these results in figure 1A have already been reported, so references should be added. Also, if there are markers (such as arrows) on the points in the figures, it would be easier to follow the context. We have replaced the images for Fig 1A to illustrate the point as best that we can. As small, but macroscopic, structures, it can be difficult to capture all elements of the fruiting body within a single focal plane, hopefully these new images give a clearer view. We have also included an additional photo of the KD mutant cells. Spore heads and enlarged basal discs have been marked as requested.

Reviewer 1: page 5, middle: it says that 'fruiting bodies with enlarged basal discs, short stalks and small spore heads (Fig. 1A)

& Reviewer 2: Figure 1 : It does not appear that the images are at the same final magnification as indicated by the scale bar - in particular image 2. The images are in fact the same magnification; gskA null mutants make smaller structures. Text has been changed to explain that fruiting bodies are also smaller

Reviewer 2: Figure 1 B: An inset of the bands for the kinase assay should be included in lieu simply adding the quantitation. Done

Figure 1C&D: These have now been re-worked and are 1C-F.

Reviewer 1: Lower panel in figure 1C: the merged images would clearly show that GSKA colocalizes to the centrosomes and nuclei. Also, the letters such as GskA-GFP, anti-butublin, and DAPI in the figures should be colored with Green, Red, and Blue, respectively. We have reorganized the Figure and included a merged image..

Reviewer 1: page 17 line 3 from the bottom: it says 'GSKA is distributed throughout the cytoplasm of wild-type cells and is in accordance with the localization of GskA-GFP.'- However, it is not true. Figure 1D shows that GskA is not evenly distributed in the cytoplasm and the merged images clearly show that GskA is not localized to the nucleus. These data conflict with those in figure 1C. We think that there may be enrichment in some parts of the cytoplasm, but we can also see the presence of some GskA in the nucleus. Text and Figures have been modified to clarify this point.

Reviewer 1: Upper panel in figure 1C is not necessary because it is repeated in the lower panel (figure 1C). &Reviewer 2: Page 5 paragraph 3 sentence 1: The authors state ".being present in both cytoplasm and nucleus, but is enriched at centrosomes (Fig. 1C)", the authors concluded that GskA-GFP is enriched at the centrosome, but use no known centrosomal marker; if the authors wish to conclude that GskA-GFP is at the centrosome, a centrosomal marker such as anti-gamma-tubulin or anti-pericentrin, as an overlay with GskA-GFP should be included. Furthermore, it is unclear why the authors show an unrepresentative image with 3 nuclei. This image, according to their Figure 4 B, only represents a small fraction of the normal phenotype, so why show something that is not representative? This is included to represent the minority of cells (<1%) that have nuclear enrichment and localization to the centrosomes. The morphology of the structures has the appearance of Dictyostelium centromeres, and common practice in Dictyostelium research is to look for the overlap with alpha-tubulin, as we have done. We have included a merged image to make this more clear. There are Dictyostelium centrosomal markers, such as CP244, gamma-tubulin and TACC (see Samereier et al., CMLS 2011), although not pericentrin. However it is not clear that these extra markers will  really add anything further to the conclusions that we already have.We have modified the text accordingly.

Reviewer 2: Page 6 paragraph 2 sentence 1: " In contrast, as cells underwent division the subcellular distribution of GskA-GFP showed clear re-localisation" . How do the authors know that this is not an artifact of the expression of GskA-GFP? We have also seen GSK-3 enriched in the nucleus using antibodies, but of course as the cells are fixed, we cannot confirm that these cells will subsequently enter mitosis. This is why one must use a GFP fusion in living cells. As we say in the text, GskA-GFP distributions match those seen using antibody. We have made slight modifications to the text to resolve this.

Reviewer 2: The authors only show 1 image of AX2 challenged with anti-GskA and state that this cell is in interphase. If the authors wish to make a point about a change in localisation of GskA at various stages of the cell cycle then anti-GskA needs to be shown for each stage of the cell cycle for which a point is made. These cell events are too few to capture sufficient numbers to get a good series by fixation and antibody staining. We have however followed these events in time-lapse. Although we do not have a full image sequence in one single video, movie 1 does show nuclear enrichment at the end of mitosis.

Reviewer 2: Furthermore, the authors state that Figure 1 D illustrates that their expression method is consistent with the normal distribution of GskA, however, there appears to be a clear difference in the nuclear localization and level of expression as measured by fluorescence of anti-GskA compared to the GskA-GFP shown in Fig 1 C. We do not observe any inconsistencies between antibody stained and GskA-GFP expressing cells. However, we have included more cells in the photographs and reduced the image exposures as they appeared a little saturated.

Figure 2

Figure 2A

Reviewer 1: The colors of GskA, Dd-EB1, and DAPI are Green, Red, and Blue, respectively. The merged image in figure 2A must be explained in the legend. The merged image has red, yellow, blue, and purple colors and there is no green from GskA-GFP. How could this happen? The images we submitted did not show this on our computers, hopefully this technical problem is now resolved with the re-submission.

Reviewer 1:page 18 line 7, 'unlike several overexpressed nuclear.. GskA-GFP enriches inside the nucleus.' However, I do not see which figures show these? Text Changed. Figures are labeled GskA-GFP.

Figure 2D

Reviewer 1: Is 'GskA-GFP is absent from kinetochores'? In my eyes, GskA-GFP colocalizes with Cenp68 since the merged image shows yellow colors as shown in the figure 2B and C indicating the colocalization of Gsk-GFP and tubulin. In order to tell the colocalization of Gsk-GFP and Cenp68, much higher resolution microscope should be used. Looking at the greyscale images, the patterns of GskA-GFP and Cenp68 look very different. However as there is a general background of Gsk-GFP in the nucleus (Dicty has a closed mitosis), we would imagine that there would be a small yellow shift on the merge. Hopefully, this is clearer on the re-submitted figures.

Figure 3

Reviewer 1: Page 8 in the middle, it says 'the assembled but unextended mitotic spindle is rarely seen in the center of the cell, and frequently shifts between focal planes, indicating that the spindle is unable to attach to the cell cortex and fix its position within the cell.'I think that authors cannot say 'indicate'. There is no data in the paper indicating the relationship between the spindle and the cell cortex. Based on the presented data in figure 3, this conclusion is totally a kind of speculation so a little mild expression instead of 'indicating' should be used.

& Reviewer 2: Page 8 paragraph 1 sentence 4: ".mitotic spindle is rarely seen in the centre of the cell, and frequently shifts between focal planes, indicating that the spindle is unable to attach to the cell cortex and fix its position." This statement and interpretation is potentially problematic. Since these cells were not immobilized the authors can not say that the spindle is moving and not the cell as a whole. The investigators need to employ either a fiducial marker and make sure that the cell is not moving as a number of cells can be seen moving in and out of the field of view. The argument that the spindle is in different focal planes is not valid. Alternatively, the control cells should be grown in suspension and shown to contain a spindle in the same focal plane throughout the experiment.

Such markers do not exist. This section has been extensively modified as requested and simply says that the unextended spindle position within the cell is not fixed.

Figure 4

Reviewer 1: Page 9 line 5, 'In wild-type cells, nuclear DNA was seen to accumulate at each of the spindle poles prior to cytokinesis. In gskA null cells, two nuclei formed and segregated to each of the spindle poles, suggesting that the process of nuclear division and chromosome segregation was not grossly affected (Fig 4C)' How could 'figure 4C' indicate the arguments like above? Please explain in more detail. This section has been modified to explain that chromosome segregation and nuclear separation occurs in both wild type and mutant cells, albeit with different kinetics

Reviewer 1: Page 9 line 10, 'but found no evidence for mis-segregation of chromosomes in the daughter cells (Fig. 4D).' The experiments of mitotic spreads were performed on the cells grown on solid plates. According to this paper, there is no defect in cytokinesis and chromosome segregation when the cells were grown in the solid plates. Therefore, in my opinion the experiments of the mitotic spreads (counting chromosomes) should be done after shaking cultures and then expressed as the number of chromosomes/nucleus/cell since gskA null cells are multinucleated in shaking cultures.

The reviewer may have misunderstood this point. There is evidence for problems of spindle separation in mutant cells grown on solid surfaces, a effect that is enhanced when cells are grown in shaking suspension leading to then becoming multinucleate. It is therefore valid to look for aneuploidy in cells growing on solid substratum. As even suspension cells would need to be plated on a slide prior to spreading, and so they would be able to re-initiate cytokinesis, it would in fact not be possible to truly examine chromosome spreads from cells truly growing in suspension. The text has been modified to clarify this point.

However, I looked at the data for Figure 4 again. Part C was in fact done with cells on a solid surface, not suspension, the figure legend was incorrect. We have replaced the original images with new ones (albeit from the same experiment). In fact we have completely remade the figure to provide a better illustration of this point and to improve the layout on the page.

Other points

Reviewer 1: gskA should be italic characters throughout the paper. *gskA* (italics) refers to the gene, GskA refers to the protein. This is a standard notation.

Reviewer 1: page 5 line 2 from the bottom, 'but is enriched at centrosomes': It would be better to explain in more detail about why you think that. Text has been modified to expand on this point.

Reviewer 1: Page 6 line6: (Data not shown) should be added at the end of the sentence. We didn’t follow this point, as the data for the kinase dead protein is shown in Fig 1B

Reviewer 1: Page 11 line 3: Space between plates and For. Done

Reviewer 1: Page 18 in the legend of fig2E, 'confocal time series taken of a mitotic wild type cell that expresses GSKA-GFP.' Should be a complete sentence. Done

Reviewer 2: Page 3 sentence 1: This statement, albeit factually correct, requires a citation. Done

Reviewer 2: Page 3 paragraph 2 sentence 2: The authors loosely use the word "inhibition" throughout the manuscript. What type of inhibition - this needs to be clarified throughout the manuscript. I.e. is this a small molecule drug, DNA/RNA approach, etc. Done – although it does make the text clunky where multiple inhibitory methods have been used.

Reviewer 2: Page 5 paragraph 1 sentence 3: This sentence needs to be clarified. Transitioning from "coordinating spindle dynamics" to "impaired cytokinesis" leaves the reader confused. Done – text simplified

Reviewer 2: Page 6 paragraph 2 sentence 8: "and remains enriched in the nuclei of daughter cells." Can you confirm that these are nuclei and not spindle microtubules depolymerizing? Dictyostelium has a closed mitosis with the nucleus remaining intact. Our image and movies show GskA-GFP filling the whole nucleus prior to and following spindle assembly and disassembly, however we agree that we cannot comment on whether this localization involves GskA-GFP interactions with microtubules, even in the absence of a fully assembled mitotic spindle. This text has now been modified.

Reviewer 2: Page 6 paragraph 2 sentence 8: movie 1 does not show what the authors say it does; Movie 1 shows spindle localization after the spindle has formed and further enrichment as division progresses. Text modified to match movie.

Reviewer 2: Page 6 paragraph 3 sentence 1: "association of GSK-3." HeLa cells have different isoforms of GSK. The authors need to clearly state if this is pan-GSK that Wakefield et al tested. Furthermore, the reviewer has evidence that GSK-3B is localized at the kinetochore region and midbody of hela cells, thus the authors overstate the agreement regarding localization of GSK. Wakefield et al do not make clear what pan-GSK3 antibody they use, but if it is from Upstate, as suggested, it is likely to be the same anti-sgg antibody used here. I don’t know how we can comment on the reviewer’s unpublished data, however we have modified the text to remove a potential “overstatement”.

Reviewer 2: Page 6 paragraph 3 sentence 1: the authors need to fine-tune the use of the word respectively in this sentence. Text modified for clarity

Reviewer 2: Figure 2 E shows a complete cell cycle in 240 seconds, however, Figure 3 A demonstrates that a complete cell cycle take 10-12 minutes. This is clearly not in agreement; it further prompts the question of how many times these experiments were conducted? The reviewers are quite right to question this point. The experiments have been repeated multiple times and by different researchers. The issue is that cell cycle times are quite variable in Dictyostelium (Uyeda, T.Q. and A. Nagasaki, Variations on a theme: the many modes of cytokinesis. Curr Opin Cell Biol, 2004. 16(1): p. 55-60). The key point is that gskA-nulls have a block in prometa-phase. Given that wild type and gskA null mutants have variation in cell cycles due to different mechanisms, it is difficult to calculate accurate differences in times between mutant and wild type. We have modified the text to expand on these points.

Figure 3: The authors are making a point about the time it takes to progress to cytokinesis. However, the authors provide little evidence that these cells started at the same stage of the cell cycle. The authors make a further point about the prometaphase stage but do not clearly show that these cells are actually at this stage. A known marker for this stage needs to be employed in replicate samples. Unfortunately, there is no marker for this stage available in Dicty, the text has been modified to take this into account.

Reviewer 2: Page 8 sentence 1: ".which is then followed by cytokinesis.(Fig. 3B, and movie 3)." These data are not in agreement. There is no evidence for cytokinesis. In fact, the videomicroscopy approach employed as movie 3 does not show cytokinesis. The text has been modified to accurately describe the movie.

Reviewer 2: Page 10 paragraph 1: " Interestingly neither of these MAPS., and we predict the presence a GSK-3." an article (i.e. the word "of") is missing between the words "presence" and "a" Text modified.

Reviewer 2: Page 10: ".co-localise at the kinetochore." there should be a citation here. Text modified.

Reviewer 2: There is no clear pattern regarding spacing between paragraphs throughout this manuscript. Done

Reviewer 2: Page 11: ".was assayed on phosphate agar plates.For.:" spacing. Done

Reviewer 2: Page 12: " 1mg/mL of freshly solved sodium..." solved? You mean dissolved? Done

Page 13: ".as described in Ryves et al, 1989. (Ryves et al." The period after 1989 belongs after the citation. Done

Reviewer 2: Page 17-19: Figure legends typically should not include materials and methods, such as fixation protocol. Furthermore, "quantification of several experiments." several tells the reader very little. It needs to be clearly stated how many independent experiments were performed. Text changed accordingly.
